# Health‐related quality of life of people with type 2 diabetes and its associated factors at a tertiary care clinic in Ningbo, China: A cross‐sectional study

**DOI:** 10.1002/edm2.353

**Published:** 2022-07-03

**Authors:** Naomi Carter, Jialin Li, Miao Xu, Li Li, Xuelan Fan, Shuyan Zhu, Pritpal Chahal, Kaushik Chattopadhyay

**Affiliations:** ^1^ Lifespan and Population Health Academic Unit, School of Medicine University of Nottingham Nottingham UK; ^2^ Department of Endocrinology and Metabolism Ningbo First Hospital Ningbo China; ^3^ Health Education England Leeds UK; ^4^ The Nottingham Centre for Evidence‐Based Healthcare: A JBI Centre of Excellence Nottingham UK

**Keywords:** diabetes mellitus, type 2, quality of life, China

## Abstract

**Introduction:**

The burden of type 2 diabetes (T2DM) in China is increasing, with potential impacts on the health‐related quality of life (HRQoL) of those who develop the disease. Context‐specific assessment of HRQoL and its associated factors informs the development of contextually appropriate interventions to improve HRQoL. This study aimed to determine the HRQoL and its associated factors in people with T2DM at a tertiary care clinic in Ningbo, China.

**Methods:**

A cross‐sectional survey was undertaken among 406 people with T2DM in 2020–21. The primary outcome was HRQoL measured using EQ VAS and EQ‐5D index from the EQ‐5D‐3L questionnaire. Multivariable regression analysis was used to determine the factors associated with HRQoL scores.

**Results:**

The mean (± standard deviation) EQ VAS score was 68.7 (13.8). Median (interquartile range) EQ‐5D index was 1 (0.027). Prevalence of problems in HRQoL domains was as follows: pain/discomfort (15.7%), anxiety/depression (13.3%), mobility (5.4%), self‐care (3.5%) and managing usual activities (5.2%). The ≥60 years age group had a mean EQ VAS score 8.7 points higher (95% CI 3.4, 13.9; *p* < .001) than the 18–39 years age group. Those with T2DM >10 years had a mean EQ VAS score 8.6 points lower than those with a duration <1 year (−12.8, −4.4; *p* = .001). A T2DM duration >10 years was associated with a reduction in the EQ‐5D index of 0.029 (−0.041, −0.016; *p* < .001) compared with a duration <1 year.

**Conclusions:**

Depression/anxiety and pain/discomfort are important domains of reduced HRQoL for this population. A longer duration of T2DM is associated with reduced HRQoL scores, including both EQ VAS and EQ‐5D index. Increasing age may be counterintuitively associated with an increase in EQ VAS score in this population, potentially reflecting a ‘paradox of aging’ process. Future work should focus on developing, evaluating and implementing interventions to improve HRQoL in T2DM, such as strategies to manage pain and mental health conditions.

## Graphical abstract

A cross‐sectional survey was undertaken among 406 people with type 2 diabetes mellitus (T2DM) in Ningbo, China, to assess their health‐related quality of life (HRQoL) and the factors associated with it. Depression/anxiety and pain/discomfort are important domains of reduced HRQoL for this population. A longer duration of T2DM is associated with reduced HRQoL scores. Increasing age may be counterintuitively associated with an increase in self rating of HRQoL in this population.

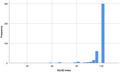

## 1 INTRODUCTION

Type 2 diabetes mellitus (T2DM) prevalence in China has risen sharply since the 1980s, with 2021 prevalence estimates of 10.6%.^1^, ^2^ The prevalence of T2DM varies across China. Eastern, coastal cities are particularly affected compared with the interior.^1^, ^3^, ^4^ Ningbo is an economically developed city in the Eastern, coastal province of Zhejiang; where T2DM prevalence exceeds 20% in the over 40s.^5^, ^6^

The impact of T2DM on morbidity is significant. In urban Chinese settings, over half of the people with T2DM have at least one chronic diabetic complication.^7^, ^8^ The condition also generates a marked economic burden, with an estimated diabetes‐related health expenditure of over 165 million US dollars in China in 2021.^2^ There has been increasing interest in the impact of T2DM on health‐related quality of life (HRQoL).^9^ HRQoL includes a person's physical, mental, emotional and social functioning.^10^

Existing evidence is indicative of T2DM reducing overall HRQoL in different settings, with a variety of potential risk factors.^11^, ^12^, ^13^, ^14^, ^15^ Importantly, HRQoL is influenced by a variety of biological, psychological, social and cultural factors and is thus context‐dependent.^16^, ^17^ Context‐specific assessment of HRQoL and its associated factors is therefore necessary, to understand how T2DM interventions can be tailored to address these factors. In Eastern China, the EQ‐5D questionnaire was used in a population‐based cross‐sectional survey across Shanghai, Jiangsu, Zhejiang and Jiangxi to measure the HRQoL of the general population and to compare this with the HRQoL of people with T2DM, who made up 5.9% of the sample.^18^, ^19^ EQ VAS is a self‐rating of overall health from 1 to 100 where 100 is perfect health. EQ‐5D index is a HRQoL summary score where 1 equates to full health, and a value of 0 equates to an HRQoL state as bad as death.^18^ People with established T2DM had a lower mean (±standard deviation [SD]) EQ VAS score than the general population (75.6 [±12.7] vs 80.1 [±11.6], *p* < .001). The same was true of mean EQ‐5D index 0.922 [±0.122] vs 0.939 [±0.111], *p* = .029). Limitations of this existing work include an absence of published data on the breakdown of scores in each of the five HRQoL domains (mobility, self‐care, usual activities, pain/discomfort and anxiety/depression). In addition, no China‐specific value sets were available at the time of the study to allow context‐specific weighting of the EQ‐5D index, therefore a value set from Japan was used instead. Factors associated with reduced EQ‐5D index in people with T2DM included female sex, lower education level, living in an area of lower economic development and increasing age.^19^ Systematic review evidence from 18 studies is suggestive of a wider range of factors associated with increased HRQoL (such as an increase in physical exercise and increase in glucose checks) and reduced HRQoL (such as the presence of complications, longer duration of T2DM, a diet with more red meat and presence of depression).^20^ The present study aimed to build on previous work to determine the HRQoL and associated factors in people with T2DM in Ningbo, by using a China‐specific value set for EQ‐5D index weighting.^21^ Through a better understanding of the factors associated with HRQoL, T2DM interventions in this setting can be tailored to address them. The present study also aimed to provide the proportion of participants reporting problems in each of the five HRQoL domains. To the best of our knowledge, this study provides the first domain‐specific breakdown of HRQoL problems in this population, which will assist in identifying potential areas for intervention.

## 2 METHODS

### 2.1 Study design, location and period

This cross‐sectional study was carried out at a tertiary care outpatient clinic in the Department of Endocrinology and Metabolism, Ningbo First Hospital, China, from 1 November, 2020, to 31 May, 2021.

### 2.2 Study population and eligibility criteria

The study included adult (18 years of age or older) patients with T2DM. The diagnosis of T2DM was based on the Chinese guideline.^22^ T2DM patients who were treated at the Ningbo First Hospital for at least 6 months prior to the survey date and gave written informed consent to participate in the study were included. T2DM duration of at least 6 months was selected to allow for patients to have time to adjust to their management at the Ningbo First Hospital.^5^

### 2.3 Sample size

At least 377 participants were required in the study based on a 95% confidence level and 5% margin of error.^23^ We approached consecutive patients with T2DM and recruited 406 participants.

### 2.4 Data collection procedures, tools and outcome measures

A survey was conducted with a quantitative questionnaire, administered in Mandarin. Data were collected and entered by a team of five trained nurses at the clinic. Another staff member was responsible for checking the data quality. The first part of the questionnaire was developed and pre‐tested among six patients with T2DM (not included in this study) and included the following variables of interest: age category (18–39 years, 40–59 years, or ≥ 60 years), sex (female or male), education level (no qualification, class 1–6, class 7–12 or college/university), occupation (manual worker, non‐manual worker or never worked/retired), residence (rural or urban as defined based on the ‘hukou’ registration system), marital status (married or single/divorced/widowed), health insurance (yes or no), duration of T2DM (≤1 year, >1–5 years, >5–10 years, >10 years), current smoker (yes or no) and current alcohol (yes or no). Data were also collected on physiological and anthropometric parameters including blood pressure (mmHg measured using a calibrated, automated sphygmomanometer), body weight (kilograms to one decimal place), height (meters to two decimal places) and waist circumference (centimetres). Each of these measurements was taken twice per participant, and the average of the two measures was used for analysis. Hypertension was defined as ≥140/90 mmHg in keeping with Chinese guidelines.^24^ Body mass index (BMI) was calculated as weight in kilograms divided by height in meters squared. Participants were categorized as ‘not overweight’ (BMI < 23), ‘overweight’ (BMI ≥ 23.0 < 27.5) or ‘obese’ (BMI ≥ 27.5) based on reference ranges specific to the Chinese population.^25^ Abdominal obesity was defined as a waist circumference of >90 cm in males or >80 cm in females based on reference ranges specific to the Chinese population.^25^ Glycosylated haemoglobin (HbA1C) was measured using venous blood sampling. Controlled diabetes was defined as HbA1C < 7% in accordance with Chinese T2DM guidelines.^22^

Data on physical activity were obtained using the validated IPAQ questionnaire (International Physical Activity Questionnaire—short form), available in Mandarin.^26^ Being physically active was defined as meeting the criteria for the moderate or high physical activity IPAQ categories.^26^ Data on diet were obtained using a modified version of the UK Diabetes and Diet Questionnaire (UKDDQ).^27^ The questions were adapted to be relevant to dietary norms in China, and the questionnaire was translated to Mandarin and pre‐tested among six patients with T2DM (not included in this study). A healthy diet was defined as having more than 50% A + B answers on the UKDDQ which correspond to ‘healthy’ dietary choices.^27^

The dependent variable HRQoL was measured using the EQ‐5D‐3L questionnaire, available in Mandarin.^18^ The EQ‐5D‐3L is a widely used generic HRQoL measurement tool which has been validated for use in T2DM.^18^, ^28^ It assesses HRQoL across five domains: mobility, self‐care, usual activities, pain/discomfort and anxiety/depression. HRQoL outcome measures obtained through EQ‐5D‐3L were (1) EQ VAS: a self‐rating between 1 and 100 of overall health, and (2) EQ‐5D index: a summary score using context‐specific value sets to attach weights to the five domains depending on how the domains are valued with respect to health. A recent EQ‐5D‐3L value set for China was used to convert the five domain health states for each participant into the weighted EQ‐5D index.^18^, ^21^ A final HRQoL outcome measure obtained was the prevalence of participants reporting no problems, some problems or extreme problems in each of the five HRQoL domains.

### 2.5 Ethics

Ethics approval was received from the Research Ethics Committee of Ningbo First Hospital (ref. 2019‐R057). The information sheet and consent form were available in Mandarin. The study objective was explained to all the eligible participants, and written informed consent was obtained from those interested in participating. They were not compelled and were free to participate in the study. They were assured regarding the anonymity, privacy, confidentiality and data protection of their information.

### 2.6 Statistical analyses

Data were entered into SPSS (SPSS IBM Statistics Version 26) for statistical analysis. Descriptive statistics were calculated including means and SDs for normally distributed continuous variables, and medians and interquartile ranges (IQR) for non‐normally distributed variables. Numbers and percentages were calculated for categorical variables. Univariate analysis to test for associations between independent variables and EQ VAS was undertaken using simple linear regression. Beta‐coefficients and 95% confidence intervals (CIs) were calculated. Multivariable analysis was undertaken using multiple linear regression via the general linear model function. Independent variables were selected for inclusion in the multivariable model using a significance cut‐off of *p* ≤ .2 in the univariate analysis. The overall significance cut‐off was set at *p* ≤ .05. Missing data were excluded listwise during multivariable regression analysis.

Due to the negative skew in the EQ‐5D index distribution, adjustments to the regression analysis for the EQ‐5D index were required due to a violation of the assumptions of linear regression. To account for this, multiple linear regression with robust standard errors was used.^29^, ^30^ Additionally, a sensitivity analysis was performed using multiple logistic regression, by converting the EQ‐5D index outcome variable into a binary variable consisting of ‘problems’ vs ‘no problems’ with HRQoL. This was used to produce odds ratios representing the odds of independent variables being associated with HRQoL problems.^29^

## 3 RESULTS

**FIGURE 1:**
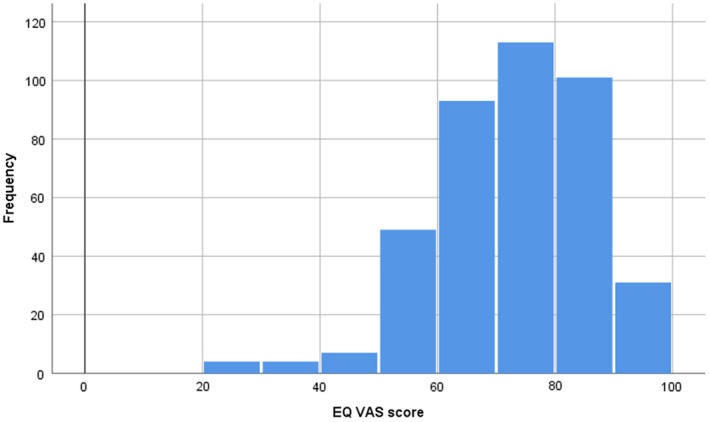
EQ‐5D‐3L VAS frequency distribution (histogram) VAS, visual analogue scale (respondent's self‐rated health on a vertical axis 0–100). Mean 68.7, SD 68.7

**FIGURE 2:**
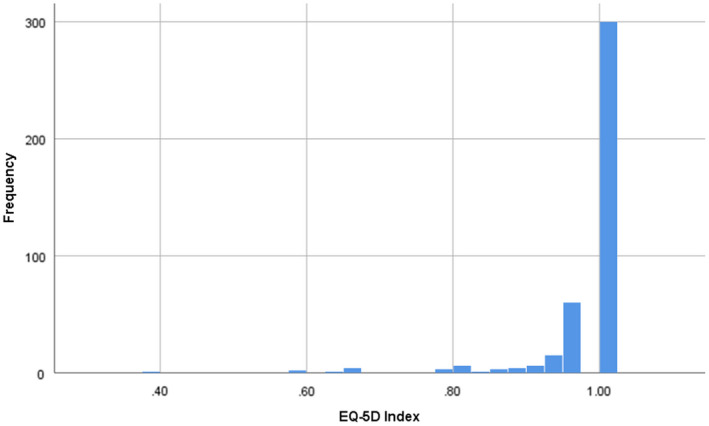
EQ‐5D index frequency distribution (histogram) Mean 0.977, standard deviation 0.065, median 1, interquartile range 0.027

During the study period, a total of 1423 patients with T2DM visited the clinic. Among these, 690 patients met the eligibility criteria. 284 eligible T2DM patients declined to participate. A total of 406 participants took part in the study. The mean (±SD) age of participants was 56.4 years (±12.6), and 62.6% were male. Further demographic, clinical and lifestyle characteristics are provided in Table 1. The mean (±SD) EQ VAS score was 68.7 (13.8), and the median (IQR) EQ‐5D index was 1 (0.027) (Table 1, Figures 1 and 2). The median and IQR were reported for the EQ‐5D index due to strong negative skew in the distribution of this variable. The overall mean (±SD) EQ‐5D index has also been calculated to allow for ease of comparison with other studies and was 0.978 (0.065). In the five domains of HRQoL, problems with pain/discomfort (15.7%) and anxiety/depression (13.3%) were followed by problems with mobility (5.4%), self‐care (3.5%) and managing usual activities (5.2%) (Table 2). 73.9% of participants reported no problems in all the five HRQoL domains (Figure 2, Table 2).

**TABLE 1:** Participant characteristics, EQ VAS and EQ‐5D index

Participant characteristics			EQ VAS			EQ‐5D index		
			(Mean ± SD)	Beta coefficient from univariate analysis (95% CI)^a^	*p* value^a^	Median (IQR)	Beta coefficient from univariate analysis (95% CI)^a^	*p* value^a^
Total population (*N*)		406	68.7 (13.8)			1 (0.027)		
Age category (*N* [%])	18–39 years	51 (12.6)	65.2 (15.4)	Ref	**.016**	1 (0)	Ref	**<.001**
	40–59 years	178 (43.8)	70.6 (13.7)	5.4 (1.2, 9.6)		1 (0)	0.004 (−0.016, 0.023)	
	≥ 60 years	174 (42.9)	67.5 (13.1)	2.3 (−1.9, 6.5)		1 (0.036)	−0.022 (−0.042, −0.003)	
	Missing	3						
Sex (*N* [%])	Female	151 (37.2)	67.3 (13.9)	Ref	.104	1 (0.036)	Ref	.075
	Male	254 (62.6)	69.6 (13.7)	2.3 (−0.5, 5.1)		1 (0)	0.012 (−0.001, 0.025)	
	Missing	1						
Education level (*N* [%])	No qualification	39 (9.6)	65.0 (12.1)	Ref	.086	1 (0.077)	Ref	.126
	Class 1–6	97 (23.9)	66.9 (13.6)	1.9 (−3.3, 7.0)		1 (0.027)	0.013 (−0.011, 0.037)	
	Class 7–12	213 (52.5)	69.9 (14.1)	4.9 (0.2, 9.6)		1 (0)	0.023 (0.001, 0.045)	
	College/university	57 (14.0)	70.0 (13.8)	5.0 (−0.6, 10.6)		1 (0)	0.025 (−0.001, 0.052)	
Occupation (*N* [%])	Manual worker	78 (19.2)	66.7 (13.6)	Ref	.264	1 (0)	Ref	**.009**
	Non‐manual worker	127 (31.3)	70.0 (15.2)	3.2 (−0.7, 7.1)		1 (0)	0.007 (−0.011, 0.025)	
	Retired or never worked	201 (49.5)	68.7 (12.9)	2.0 (−1.7, 5.6)		1 (0.027)	−0.015 (−0.031, 0.002)	
Residence (*N* [%])	Urban	251 (61.8)	69.2 (13.7)	Ref	.305	1 (0.027)	Ref	.078
	Rural	154 (37.9)	67.9 (14.1)	−1.4 (−4.2, 1.3)		1 (0.027)	−0.012 (−0.025, 0.001)	
	Missing	1						
Marital status (*N* [%])	Married	384 (94.6)	68.8 (13.8)	Ref	.584	1 (0.027)	Ref	.507
	Single/widowed/divorced	22 (5.4)	68.0 (14.0)	−1.6 (−7.5, 4.2)		1 (0.036)	−0.009 (−0.036, 0.018)	
Health insurance (*N* [%])	Yes	389 (95.8)	68.7 (13.9)	Ref	.641	1 (0.027)	Ref	.443
	No	17 (4.2)	68.2 (10.9)	−1.6 (−8.1, 5.0)		1 (0.032)	0.012 (−0.019, 0.430)	
Duration of T2DM in years (*N* [%])	≤1	70 (17.2)	73.5 (12.9)	Ref	**.003**	1 (0)	Ref	**.001**
	>1–5	88 (21.7)	68.4 (15.5)	−5.1 (−9.4, −0.9)		1 (0)	−0.012 (−0.032, 0.008)	
	>5–10	85 (20.9)	69.6 (12.7)	−3.9 (−8.2, 0.4)		1 (0.027)	−0.018 (−0.038, 0.002)	
	>10	163 (40.1)	66.4 (13.3)	−7.2 (−11.0, −3.3)		1 (0.036)	−0.035 (−0.052, −0.017)	
Current smoker (*N* [%])	No	312 (76.8)	68.7 (13.7)	Ref	.875	1 (0.027)	Ref	.075
	Yes	93 (22.9)	68.4 (14.0)	−0.3 (−3.4, 2.9)		1 (0)	0.013 (−0.001, 0.027)	
	Missing	1						
Current alcohol (*N* [%])	No	288 (70.9)	68.7 (13.4)	Ref	.960	1 (0.027)	Ref	**.045**
	Yes	118 (29.1)	68.7 (14.8)	−0.1 (−3.0, 3.0)		1 (0)	0.014 (0, 0.028)	
Body mass index (BMI) (*N* [%])	Not overweight (<23)	135 (33.3)	68.0 (13.9)	Ref	.468	1 (0.027)	Ref	.456
	Overweight (≥23.0 < 27.5)	194 (47.8)	68.5 (14.2)	0.5 (−2.6, 3.5)		1 (0.027)	0.007 (−0.007, 0.029)	
	Obese (≥27.5)	76 (18.7)	70.1 (12.4)	2.4 (−1.5, 6.2)		1 (0)	0.011 (−0.007, 0.029)	
	Missing	1						
Abdominal obesity (*N* [%])	No	152 (37.4)	67.8 (14.1)	Ref	.399	1 (0.027)	Ref	.834
	Yes	245 (60.3)	69.0 (13.8)	1.2 (−1.6, 4.0)		1 (0.027)	0.001 (−0.012, 0.015)	
	Missing	9						
Hypertension (*N* [%])	No	295 (72.7)	68.4 (13.9)	Ref	.471	1 (0)	Ref	.177
	Yes	111 (27.3)	69.4 (13.6)	−1.1 (−4.1, 1.9)		1 (0.027)	0.010 (−0.004, 0.024)	
HbA1C (*N* [%])	Controlled (<7)	114 (28.1)	70.1 (14.4)	Ref	.196	1 (0.007)	Ref	.530
	Uncontrolled (≥7)	286 (70.4)	68.2 (13.6)	−2.0 (−5.0, 1.0)		1 (0.027)	0.004 (−0.009, 0.018)	
	Missing	6						
Physically active (*N* [%])	No	73 (18.0)	67.2 (13.0)	Ref	.301	1 (0.014)	Ref	.110
	Yes	333 (82.0)	69.0 (14.0)	1.8 (−1.6, 5.3)		1 (0.027)	0.013 (−0.003, 0.030)	
Healthy dietary habits (*N* [%])	No	8 (2.0)	70.6 (14.7)	Ref	.692	0.986 (0.034)	Ref	.773
	Yes	396 (97.5)	68.7 (13.8)	−2.0 (−11.7, 7.8)		1 (0.027)	−0.007 (−0.052, 0.039)	
	Missing	2						

Abbreviations: EQ VAS, EQ‐5D visual analogue scale (respondent's self‐rated health on a vertical axis 0–100); EQ‐5D index, summary index value of health states; *N*, number; SD, standard deviation; IQR, interquartile range; kg, kilogram; m, meters; CI, confidence interval; Ref, reference category; T2DM, type 2 diabetes mellitus.

^a^ Simple linear regression using the general linear model in SPSS. Significant results highlighted in bold.

**TABLE 2:** EQ‐5D‐3L frequencies reported by dimension and level

	Mobility *N* (%)	Self‐Care *N* (%)	Usual activities *N* (%)	Pain/discomfort *N* (%)	Anxiety/depression *N* (%)
Level 1 (no problems)	384 (94.6)	392 (96.6)	385 (94.8)	342 (84.2)	352 (86.7)
Level 2 (some problems)	21 (5.2)	6 (1.5)	19 (4.7)	59 (14.5)	53 (13.1)
Level 3 (extreme problems)	1 (0.2)	8 (2.0)	2 (0.5)	5 (1.2)	1 (0.2)
Total	406	406	406	406	406

Abbreviation: *N*, number.

In the univariate analysis (Table 1), significant associations were found between EQ VAS score and age category (*p* = .016) and duration of T2DM (*p* = .003). Significant associations were also found between EQ‐5D index and age category (*p* < .001), occupation (*p* = .009), duration of T2DM (*p* = .001) and being a current alcohol drinker (*p* = .045). In the multivariable analysis, the association between both age category and duration of T2DM with EQ VAS remained significant (Table 3). The ≥60 years age group had a mean EQ VAS score 8.7 points higher (95% CI 3.4, 13.9; *p* < .001) than the 18–39 years reference age group. Those with T2DM for over 10 years had a mean EQ VAS score on average 8.6 points lower than those with T2DM for under 1 year (−12.8, −4.4; *p* = .001). The findings were adjusted for the potential confounding factors of sex, education level and control of T2DM using multiple linear regression.

**TABLE 3:** Factors associated with EQ VAS assessed by multiple linear regression

Participant characteristics		Association with EQ VAS (beta‐coefficient from multivariate analysis [95% CI])	*p* value
**Age category (years)**	**18–39** **40–59** **≥60**	**Ref** **8.5 (3.9, 13.0)** **8.7 (3.4, 13.9)**	**.001**
Sex	Female Male	Ref 0.8 (−2.2, 3.8)	.587
Education level	No qualification Class 1–6 Class 7–12 College/university	Ref 0.5 (−4.7, 5.7) 4.3 (−0.8, 9.4) 5.7 (−0.8, 12.1)	.081
**Duration of T2DM (years)**	**≤1** **>1–5** **>5–10** **>10**	**Ref** **−5.1 (−9.5, −0.8)** **−4.5 (−9.0, −0.1)** **−8.6 (−12.8, −4.4)**	**.001**
HbA1C (%)	Controlled (<7) Uncontrolled (≥7)	Ref −1.6 (−4.6, 1.4)	.306

*Note*: Multiple linear regression undertaken in SPSS using the general linear model. Independent variable selection using *p* ≤ .2 in univariate analysis. Adjusted *R*‐squared = 0.058. Multicollinearity excluded with cut‐off for variance inflation factor (VIF) of <5. Significant associations highlighted in bold.

Abbreviations: EQ VAS, EQ‐5D visual analogue scale (respondent’s self‐rated health on a vertical axis 0‐100); EQ‐5D index, summary index value of health states; CI, confidence interval; Ref, reference category; T2DM, type 2 diabetes mellitus.

In the multivariable analysis looking at factors associated with the EQ‐5D index, only the duration of T2DM was significant (Table 4). A T2DM duration of over 10 years was associated with a reduction in the EQ‐5D index of 0.029 (−0.041, −0.016; *p* < .001) compared with those with T2DM for ≤1 year. Smaller but significant reductions in EQ‐5D index were seen with T2DM durations of 5–10 years (−0.017 [−0.032, −0.002]; *p* = .024) and 1–5 years (−0.013 [−0.035, −0.001]; *p* = .040) compared with the reference category of duration <1 year, indicative of a dose–response relationship. The results were adjusted for age, sex, educational level, occupation, residence, smoking status, alcohol status, presence of hypertension and being physically active or not using multiple linear regression.

**TABLE 4:** Factors associated with EQ‐5D index assessed by multiple linear regression with robust standard errors

Participant characteristics		Association with EQ‐5D index (beta‐coefficient from multivariate analysis [95% CI))	*p* value
Age category (Years)	18–39 40–59 ≥60	Ref 0.009 (−0.005, 0.022) −0.004 (−0.020, 0.013)	Ref .203 .658
Sex	Female Male	Ref 0.004 (−0.014, 0.022)	Ref .676
Education level	No qualification Class 1–6 Class 7–12 College/university	Ref 0.003 (−0.025, 0.031) 0.009 (−0.019, 0.036) 0.001 (−0.030, 0.032)	Ref .824 .535 .939
Occupation	Manual worker Non‐manual worker Retired or never worked	Ref 0.002 (−0.008, 0.013) 0.003 (−0.015, 0.020)	Ref .673 .767
Residence	Urban Rural	Ref 0.010 (−0.005, 0.025)	Ref .193
**Duration of T2DM (years)**	≤1 **>1–5** **>5–10** **>10**	Ref **−0.013 (−0.035, −0.001)** **−0.017 (−0.032, −0.002)** **−0.029 (−0.041, −0.016)**	Ref **.040** **.024** **<.001**
Current smoker	No Yes	Ref 0 (−0.013, 0.130)	Ref .981
Current alcohol	No Yes	Ref −0.007 (−0.020, 0.005)	Ref .269
Hypertension	No Yes	Ref 0.010 (−0.007, 0.027)	Ref .250
Physically active	No Yes	Ref 0.016 (−0.008, 0.041)	Ref .198

*Note*: Multiple linear regression undertaken in SPSS using the general linear model with robust standard errors due to the non‐normal distribution of the dependent variable. Independent variable selection using *p* ≤ 0.2 in univariate analysis. Adjusted *R*‐squared = 0.047. Multicollinearity excluded with cut‐off for variance inflation factor (VIF) of <5. Significant associations highlighted in bold.

Abbreviations: EQ‐5D index, summary index value of health states; CI, confidence interval; Ref, reference category; T2DM, type 2 diabetes mellitus.

In the sensitivity analysis, T2DM duration of over 10 years and 5–10 years, both remained significantly associated with HRQoL problems (OR 6.77, [95% CI 2.45, 18.74]; *p* < .001 and 5.56, [1.98, 15.63]; *p* = .001, respectively). Being a current alcohol drinker was also significantly associated with reduced odds of HRQoL problems in the sensitivity analysis (0.45, [0.2, 0.84]; *p* = .13). The remainder of the independent variables did not demonstrate any significant associations in the sensitivity analysis.

## 4 DISCUSSION

The first objective of the study was to determine the HRQoL in people with T2DM attending a tertiary care clinic in Ningbo, China. The mean (±SD) EQ VAS score in this population was 68.7 (13.8). This is lower than the mean EQ VAS score in a cross‐sectional study of people with T2DM across a wider area of Eastern China, which was 75.6 (12.7).^19^ Conversely, the present study demonstrated very high EQ‐5D index scores, with a median of 1 (IQR 0.027) and a mean of 0.978 (SD 0.065). This reflects nearly three‐quarters (73.9%) of participants reporting no problems in all five HRQoL domains. This strong negative skew in EQ‐5D index is commonly seen, however, is more marked in the present study than in previous literature assessing the EQ‐5D index in the general population of China, as well as in those with T2DM and other chronic conditions in both China and neighbouring Vietnam.^19^, ^31^, ^32^, ^33^ The EQ VAS is a broader concept than the domain‐specific problem rating of the EQ‐5D index, and it may capture aspects of HRQoL that are not represented in EQ‐5D domains.^34^

Areas in which problems were most frequently reported with HRQoL were pain/discomfort (15.7%) and anxiety/depression (13.3%). Attention should be paid to screening and intervention for painful complications of T2DM such as peripheral neuropathy, which have previously been proven to be common in this population.^8^, ^35^ The high prevalence of psychological distress found is in keeping with systematic review evidence demonstrating a high prevalence of depressive symptoms (37.8% [95% CI 34.6–41.0) and anxiety symptoms (28.9% [21.0–36.9] in people with T2DM living in China.^36^ Even higher rates of depressive symptoms have been found in elderly patients living with T2DM in neighbouring Vietnam (79.4%).^37^ A previous study conducted by the team found mental health conditions to be uncommon in the T2DM population in Ningbo, noting the risk of under‐reporting.^38^ The present study suggests that problems with anxiety and depression are in fact relatively common in this population, which can be the case even when a formal diagnosis is lacking. The interplay between chronic diseases such as diabetes and mental health is complex. Chronic disease burden is able to negatively impact mental health, whilst poor mental health can also worsen chronic disease outcomes.^39^, ^40^ The findings are suggestive of the importance of screening and intervention for mental health conditions in people with T2DM in this setting. Community‐ and family‐based interventions are common approaches to addressing HRQoL in T2DM and could be specifically tailored to address mental health aspects of care.^41^

The second objective of the study was to determine the factors associated with HRQoL in this population with T2DM. A longer duration of T2DM was found to be significantly associated with reduced EQ VAS score and EQ‐5D index. This is in keeping with previous work which found HRQoL in those with newly diagnosed T2DM was comparable with the general population, whilst that of people with established T2DM was reduced.^19^ Duration of T2DM has also been linked to HRQoL in other study settings.^20^, ^42^, ^43^ In contrast to this, a further study found no association between the duration of T2DM and HRQoL.^12^ It may be important to consider the role of the accumulation of comorbidities and complications over time as a possible confounding factor.^14^ Whilst the duration of the disease itself is not mitigatable, the risk of complications can be reduced through good glycaemic control which may reduce the risk of declining HRQoL over time.^44^

Interestingly, the EQ VAS score was shown to increase with increasing age. This contrasts with existing research suggestive of worsening HRQoL in T2DM with increasing age.^11^, ^19^, ^20^ This effect was not seen on EQ‐5D index, where no significant association with age was found. The ‘paradox of aging’ describes a phenomenon of maintenance or even improvement of subjective HRQoL with increasing age. This runs in parallel with objective reductions in physical HRQoL, with a weakening of the association between subjective and objective HRQoL with progressive age.^45^ For example, evidence from a longitudinal study in Taiwan demonstrated a positive correlation between increasing age and mental HRQoL scores, and a negative correlation between age and physical HRQoL scores.^45^ It is possible that the subjective, self‐rating EQ VAS score is reflecting this ‘paradox of aging’ process.

A significant association was seen between current alcohol drinking and HRQoL problems in the sensitivity analysis, with reduced odds of HRQoL problems in current alcohol drinkers compared with those who did not currently drink alcohol. This association was not seen in relation to the EQ VAS score or the EQ‐5D index in the primary analyses. Moderate alcohol drinking has been linked to higher initial HRQoL scores in some populations although with a decline over time.^46^ An alternative explanation is that the effect of alcohol drinking on HRQoL is being confounded by additional factors such as socioeconomic status.^47^

Strengths of the study included the use of a China‐specific value set for the calculation of the EQ‐5D index, as well as generally low rates of missing data (Table 1). To the best of our knowledge, this study also provided the first domain‐specific breakdown of HRQoL problems in this population. Caution should be applied in generalizing the results of this study to other settings due to the context‐specific nature of HRQoL. A qualitative exploration of the HRQoL impacts of T2DM in this context would be beneficial for a richer understanding of the experience of people with T2DM. It is important to consider the clinical context in which HRQoL scores are measured. For example, higher levels of comorbidity and subsequent HRQoL problems might be expected in tertiary clinic populations versus community settings. There were also some other limitations. The multiple linear regression models had adjusted *R* squared values of 0.058 and 0.047 for the EQ VAS model and the EQ‐5D index model, respectively. This suggests that the factors in the model explain a relatively small amount of the HRQoL variation in this population. There are likely to be additional unknown factors influencing HRQoL which were not included in the analysis, such as comorbidities and complications of T2DM beyond obesity and hypertension.^48^ Evidence from China suggests that the number of simultaneous diseases someone suffers from is correlated with HRQoL scores, indicative of a cumulative burden effect.^49^ Reported rates of a healthy diet (97.5%) and adequate physical activity (82.0%) were also very high in the present study which may be indicative of some social desirability bias in participant questionnaire completion. This could mask a relationship between poor diet or reduced physical activity and HRQoL.

In conclusion, the current study has demonstrated that depression/anxiety and pain/discomfort are important areas of reduced HRQoL for patients with T2DM in a tertiary clinic in Ningbo, China. Duration of T2DM is a risk factor for reduced HRQoL scores in this population. Increasing age may be counterintuitively associated with an increase in EQ VAS score in this population. Future work should focus on assessing interventions to improve HRQoL in T2DM, such as strategies to manage pain and mental health conditions.

## AUTHOR CONTRIBUTIONS

**Naomi Carter:** Formal analysis (lead); methodology (supporting); visualization (equal); writing – original draft (lead); writing – review and editing (equal). **Jialin Li:** Conceptualization (equal); data curation (equal); funding acquisition (equal); investigation (equal); methodology (equal); project administration (equal); supervision (equal); writing – review and editing (equal). **Miao Xu:** Conceptualization (equal); data curation (equal); funding acquisition (equal); investigation (equal); methodology (equal); project administration (equal); supervision (equal); writing – review and editing (equal). **Li Li:** Conceptualization (equal); data curation (equal); funding acquisition (equal); investigation (equal); methodology (equal); project administration (equal); supervision (equal); writing – review and editing (equal). **Xuelan Fan:** Data curation (equal); investigation (equal); project administration (equal); writing – review and editing (equal). **Shuyan Zhu:** Data curation (equal); investigation (equal); project administration (equal); writing – review and editing (equal). **Pritpal Chahal:** Supervision (equal); writing – review and editing (equal). **Kaushik Chattopadhyay:** Conceptualization (equal); formal analysis (supporting); methodology (equal); project administration (equal); supervision (lead); visualization (equal); writing – original draft (supporting); writing – review and editing (equal).

## CONFLICT OF INTEREST

None known.

## ETHICAL STATEMENT

Ethics approval was received from the Research Ethics Committee of Ningbo First Hospital (ref. 2019‐R057).

## Data Availability

The dataset will be available upon request unless there are legal or ethical reasons for not doing so.
